# Gelatin Hydrolysate from Bigeye Snapper (*Priacanthus tayenus*) Skin Attenuates the Progression of Diabetic Nephropathy in Rats by Modulating Oxidative Stress, Inflammatory Responses, and Endoplasmic Reticulum Stress Signaling Pathways

**DOI:** 10.3390/ijms27177682

**Published:** 2026-08-27

**Authors:** Krit Jaikumkao, Khin Thandar Htun, Nattavadee Pengrattanachot, Prempree Sutthasupha, Sasivimon Promsan, Napatsorn Montha, Onanong Jaruan, Rarin Suriyalungka, Phassaraporn Khadtha, Sompong Sriburee, Suchart Kothan, Sutee Wangtueai, Anusorn Lungkaphin

**Affiliations:** 1 Department of Radiologic Technology, Faculty of Associated Medical Sciences, Chiang Mai University, Chiang Mai 50200, Thailand; krit.ja@cmu.ac.th (K.J.); ssearrin@gmail.com (R.S.); patsarapron0901@gmail.com (P.K.); sompong.sriburee@cmu.ac.th (S.S.); suchart.kothan@cmu.ac.th (S.K.); 2 Center of Radiation Research and Medical Imaging, Department of Radiologic Technology, Faculty of Associated Medical Sciences, Chiang Mai University, Chiang Mai 50200, Thailand; ktdhtun28@gmail.com; 3 Department of Physiology, Faculty of Medicine, Kasetsart University, Bangkok 10900, Thailand; ntvd.peng@gmail.com; 4 Renal Transporter and Molecular Signaling Unit, Department of Physiology, Faculty of Medicine, Chiang Mai University, Chiang Mai 50200, Thailand; prempree.sut@cmu.ac.th (P.S.); o.jaruan@gmail.com (O.J.); 5 Division of Physiology, School of Medical Sciences, University of Phayao, Phayao 56000, Thailand; sasivimon.ps@gmail.com; 6 Department of Animal and Aquatic Science, Faculty of Agriculture, Chiang Mai University, Chiang Mai 50200, Thailand; napatsorn.m@cmu.ac.th; 7 School of Agro-Industry, Faculty of Agro-Industry, Chiang Mai University, Chiang Mai 50100, Thailand; sutee.w@cmu.ac.th; 8 Cluster of Innovation for Sustainable Seafood Industry and Value Chain Management, Chiang Mai University, Chiang Mai 50200, Thailand; 9 Functional Foods for Health and Disease, Department of Physiology, Faculty of Medicine, Chiang Mai University, Chiang Mai 50200, Thailand

**Keywords:** diabetic nephropathy, fish gelatin hydrolysate, oxidative stress, inflammation, fibrosis, ER stress

## Abstract

Diabetic nephropathy (DN) is a major complication of chronic hyperglycemia. Although fish gelatin hydrolysate has antioxidant, anti-inflammatory, and antihypertensive properties, its renoprotective effects in DN remain unclear. This study investigated its effects on renal oxidative stress, inflammation, fibrosis, and endoplasmic reticulum (ER) stress in diabetic rats. Thirty male Wistar rats were fed a normal chow diet or a high-fat diet for 4 weeks. Diabetes was induced in high-fat-diet-fed rats by a single intraperitoneal injection of streptozotocin (30 mg/kg). Diabetic rats were randomly assigned to receive no treatment (untreated diabetes), gelatin hydrolysate at 250 or 500 mg/day, or metformin at 100 mg/kg/day groups (*n* = 6/group), while normal-chow-fed rats served as controls. Treatments were orally administered for 8 weeks. High-dose gelatin hydrolysate improved metabolic disturbances and renal dysfunction, whereas low-dose gelatin hydrolysate and metformin more effectively reduced oxidative stress, as indicated by decreased 8-OHdG expression. All treatments reduced renal injury, fibrosis, and inflammatory markers. ER stress-related proteins were also attenuated, with the greatest changes observed in IRE1/XBP1-associated markers. These findings provide in vivo evidence that GLH treatment is associated with improved renal outcomes and reduced markers of oxidative stress, inflammation, fibrosis, and ER stress in DM rats.

## 1. Introduction

With the increase in global prevalence of diabetes mellitus (DM), diabetic kidney disease (DKD) anddiabetic nephropathy (DN), remain the leading cause of chronic kidney disease (CKD) [1]. DN is a critical microvascular complication affecting individuals with DM. It initially manifests as hyperglycemia, proteinuria, and reduced glomerular filtration rate (GFR). As DN progresses, it is marked by an increase in the extracellular matrix (ECM), a reduction in glomerular podocytes, mesangial hypertrophy, and thickening of glomerular basement membranes. These pathological changes culminate in glomerulosclerosis and eventual kidney failure [2]. DN can develop in both type 1 diabetes mellitus (T1DM) and type 2 diabetes mellitus (T2DM), although their underlying metabolic characteristics differ. T1DM is primarily characterized by absolute insulin deficiency, whereas T2DM involves insulin resistance and progressive pancreatic β-cell dysfunction and is frequently accompanied by obesity, dyslipidemia, and hypertension [3,4]. However, chronic hyperglycemia, hypertension, genetic susceptibility, and metabolic and hemodynamic stress contribute to renal injury in both types of diabetes [5]. DN screening generally begins five years after T1DM diagnosis, whereas patients with T2DM are screened at diagnosis because hyperglycemia has been present for several years before the disease is clinically recognized [6].

Chronic hyperglycemia induces kidney toxicity by promoting the formation of advanced glycation end products (AGEs), which accumulate in renal tissue and trigger oxidative stress and inflammation [7]. Elevated glucose also activates protein kinase C (PKC), transforming growth factor-beta (TGF-β), and renin–angiotensin–aldosterone system (RAAS) pathways, leading to cell proliferation, hypertrophy, fibrosis, and inflammation [8]. Reactive oxygen species (ROS) and cytokines further worsen endothelial dysfunction and podocyte injury [9]. The pathogenesis of DN is multifactorial, highlighting the need to understand these mechanisms for developing effective therapeutic strategies.

In metabolic disorders, such as diabetes, the accumulation of misfolded and unfolded proteins triggers endoplasmic reticulum (ER) stress, which activates the unfolded protein response (UPR) to counter this stress [10]. The UPR employs its effectors, PRKR-like ER kinase (PERK), inositol-requiring enzyme 1α (IRE1α), and activating transcription factor 6 (ATF6), to halt protein translation and enhance the folding capacity of the ER, thereby promoting cell survival [11]. However, prolonged ER stress leads to a maladaptive UPR characterized by increased transcription of C/EBP homologous protein (CHOP), initiating apoptotic signaling in affected cells and contributing to organ dysfunction. Understanding the intricate relationship between ER stress and DN is crucial for developing targeted therapeutic strategies aimed at alleviating ER stress and mitigating the progression of DN.

Controlling blood glucose and blood pressure through medication might prevent or delay kidney issues and other complications [12]. However, several medications not only exert the benefit of treating the disease but also may induce some serious adverse effects. Overuse or inappropriate management of drugs may result in significant adverse effects, which may lead to patient suffering and affect their quality of life.

Fish gelatin hydrolysate (GLH) is derived from fish gelatin, which is a protein extracted from collagen-rich tissues of fish byproducts such as skin, scales, and bones [13]. Because of its functional properties, including gelling, thickening, and stabilizing abilities, it is commonly utilized in various food and pharmaceutical applications [14]. Consequently, it is increasingly being investigated for use in functional foods, dietary supplements, and biomedical applications. The hydrolysis process in fish gelatin involves the breaking down of proteins into smaller peptides or amino acids using enzymatic or chemical methods [15]. This hydrolyzed fish gelatin offers several advantages over intact gelatin, such as enhanced functional properties, easier digestion, and improved bioactivities [15].

Gelatin-derived peptides have attracted interest as potential adjunctive candidates for diabetes and its complications. Protein hydrolysates and bioactive peptides have demonstrated antioxidant [16,17], anti-inflammatory [18,19], and ACE-inhibitory properties [20,21,22], which may be relevant to the pathological processes underlying DN. GLH from bigeye snapper skin has also shown protective effects against oxidative stress-related tissue injury in preclinical studies [23], while gelatin-derived peptides from fish and porcine sources have been reported to improve glycemic control, possibly through DPP-4 inhibition and enhanced GLP-1 activity [24,25]. These findings suggest that gelatin-derived peptides may influence both metabolic disturbances and cellular stress associated with diabetes.

The nephroprotective effects of GLH in DN remain unexplored. This study investigated its protective role and mechanisms in DN rats, focusing on oxidative stress, inflammation, fibrosis, and ER stress. We hypothesized that GLH mitigates DN by suppressing these pathways. Metformin (100 mg/kg/day) was used as a positive control, based on its known metabolic and renoprotective benefits in diabetic conditions [26].

## 2. Results

### 2.1. The Effect of GLH on Metabolic Parameters in Rats with Diabetes

**Figure 1:**
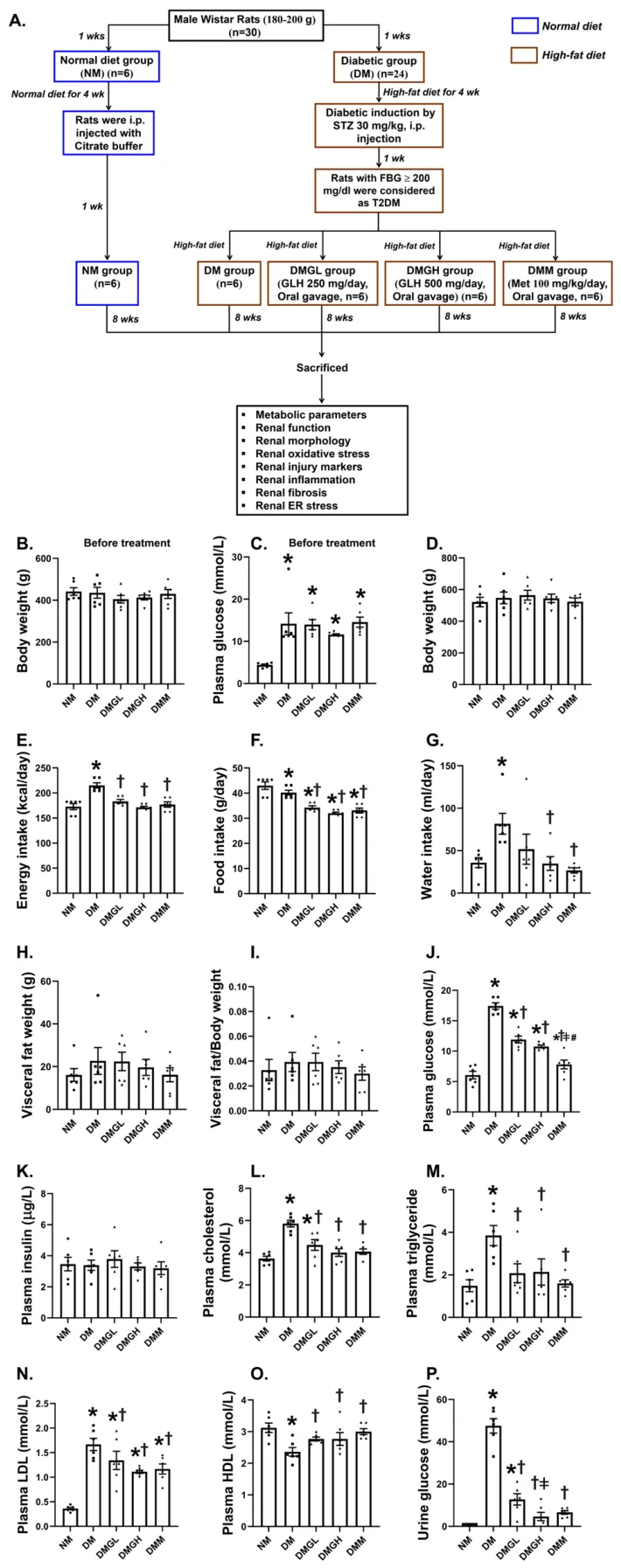
Experimental timeline (**A**); baseline body weight (**B**) and plasma glucose levels (**C**) before treatment; and the effects of fish gelatin hydrolysate (GLH) on body weight (**D**), energy intake (**E**), food intake (**F**), water intake (**G**), visceral fat weight (**H**), visceral fat-to-body weight ratio (**I**), plasma glucose (**J**), plasma insulin (**K**), plasma total cholesterol (**L**), plasma triglycerides (**M**), plasma LDL (**N**), plasma HDL (**O**), and urine glucose (**P**) in diabetic (DM) rats. Data are presented as mean ± SEM; (*n* = 6 rats/group). NM: normal control; DM: diabetes; DMGL: diabetic plus GLH (low dose: 250 mg); DMGH: diabetic plus GLH (high dose: 500 mg); DMM: diabetic plus metformin. Data are presented as mean ± standard error of the mean (SEM, *n* = 6 per group). * *p* < 0.05 vs. NM, ^†^ *p* < 0.05 vs. DM, ^‡^ *p* < 0.05 vs. DMGL, ^#^ *p* < 0.05 vs. DMGH. Individual data points are represented by circles (NM), squares (DM), upward triangles (DMGL), downward triangles (DMGH), and diamonds (DMM).

In this study, we aimed to establish a DM model in rats. Blood glucose levels were significantly increased, surpassing 11.1 mmol/L, in DM rats compared to those in normal rats (*p* < 0.05) (Figure 1C). However, there was no difference in body weight among the groups (Figure 1B). In DM rats, energy and water intake, plasma levels of glucose, cholesterol, triglycerides, LDL, and urine glucose significantly increased in comparison to those in the normal group (*p* < 0.05) (Figure 1E,G,J,L–N,P). However, there were no changes in body weight, visceral fat weight, visceral fat weight/body weight ratio, or plasma insulin levels across experimental groups (Figure 1D,H,I,K). On the contrary, the DMGL, DMGH, and DMM groups exhibited a significant reduction in food intake, as well as reduced plasma levels of glucose, cholesterol, triglycerides, LDL, and urine glucose compared to the DM group (*p* < 0.05). Additionally, DMGH and DMM showed significantly reduced water intake (*p* < 0.05), but this effect was not observed in the DMGL group. The DMGH group also showed significantly lower plasma cholesterol and urine glucose levels than the NM group and the DMGL group (*p* < 0.05), respectively. However, a significant decrease in plasma glucose levels was observed in the DMM group compared to those in the DM, DMGL, and DMGH groups (*p* < 0.05). It is noteworthy that plasma HDL levels in the DM group were lower than those in normal rats (*p* < 0.05). Furthermore, the DMGL, DMGH, and DMM groups had significantly increased plasma HDL levels compared to the DM group (*p* < 0.05). These results indicate that all treatments improved metabolic parameters compared with the untreated DM group. However, metformin produced a significantly greater reduction in plasma glucose than either low- or high-dose GLH.

### 2.2. The Effect of GLH on Renal Function, Renal Lipid Accumulation and Renal Pathological Changes in Rats with Diabetes

**Figure 2:**
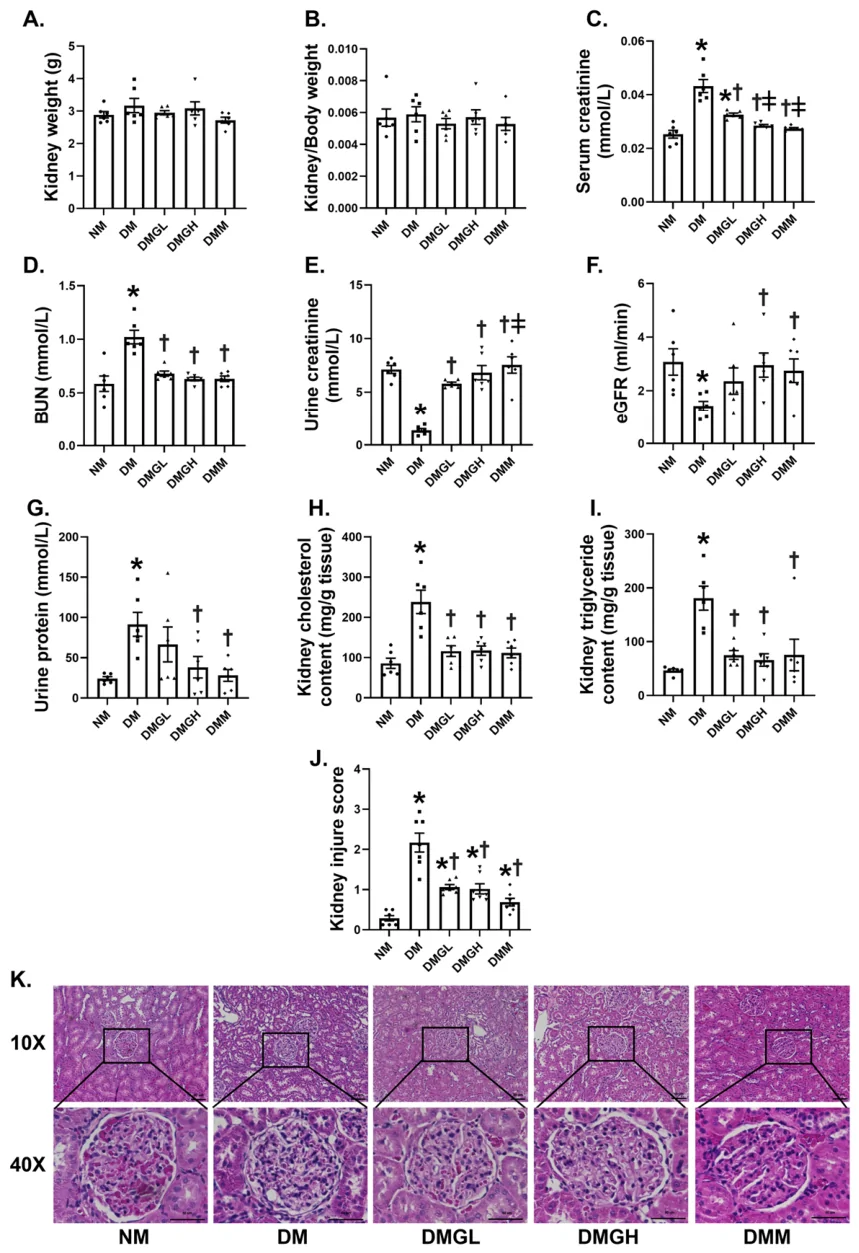
Effects of GLH on metabolic parameters and kidney injury and function. Effects of GLH on kidney weight (**A**), kidney/body weight (**B**), serum creatinine (**C**), serum BUN (**D**), urine creatinine (**E**), eGFR (**F**), urine protein (**G**), kidney cholesterol content (**H**), kidney triglyceride content (**I**), kidney injury score (**J**), and kidney H&E stain (**K**) in DM rats. NM: normal control; DM: diabetes; DMGL: diabetic plus GLH (low dose: 250 mg); DMGH: diabetic plus GLH (high dose: 500 mg); DMM: diabetic plus metformin. Data are presented as mean ± standard error of the mean (SEM, *n* = 6 per group). * *p* < 0.05 vs. NM, ^†^ *p* < 0.05 vs. DM, ^‡^ *p* < 0.05 vs. DMGL. Individual data points are represented by circles (NM), squares (DM), upward triangles (DMGL), downward triangles (DMGH), and diamonds (DMM). Scale bar = 50 μm.

As shown in Figure 2C,G–K, the DM group exhibited significantly higher levels of serum creatinine, BUN, urine protein, kidney lipid content, and kidney injury score than the NM group (*p* < 0.05). Notably, urine protein levels were significantly reduced in both the DMGH and DMM groups (*p* < 0.05), showing a functional improvement that closely parallels the changes observed in GFR. Concurrently, these DM rats showed notably lower levels of urine creatinine and eGFR relative to the NM group (*p* < 0.05), indicating typical features of DN (Figure 2E,F). There were no significant differences in kidney weight or kidney weight/body weight among the groups (Figure 2A,B). Following 8 weeks of treatment with GLH (250 or 500 mg), the levels of these kidney injury biomarkers improved compared to those in the DM group (*p* < 0.05). The positive control, i.e., metformin, significantly ameliorated all parameters of kidney damage (*p* < 0.05), demonstrating similar efficacy to high-dose GLH. Importantly, the DMGH group exhibited significantly lower serum creatinine (*p* < 0.05) and slightly higher urine creatinine levels compared to the DMGL group. Treatment with high-dose GLH or metformin was associated with improvements in several renal functional parameters in diabetic rats.

### 2.3. The Effect of GLH on Renal Oxidative Stress in Rats with Diabetes

**Figure 3:**
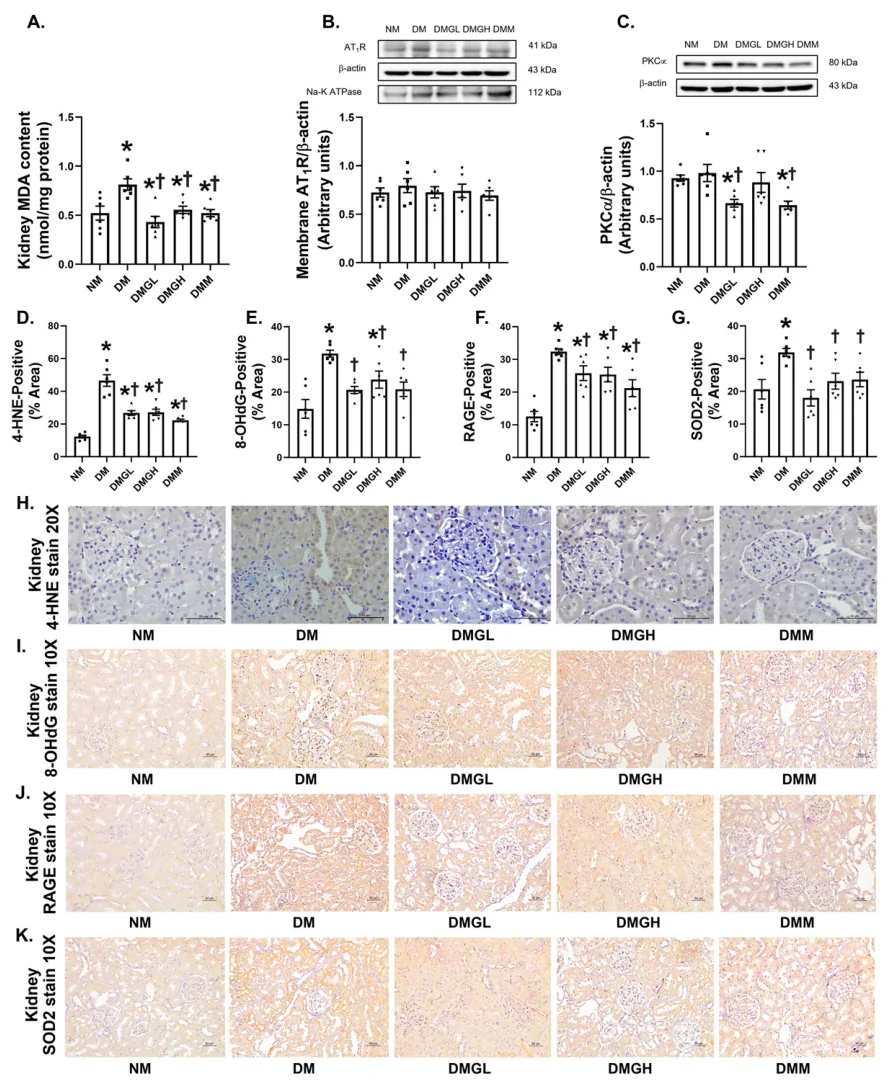
Effects of GLH on renal oxidative stress. Effects of GLH on kidney MDA content (**A**), kidney expression of AT_1_R (**B**), PKCα (**C**), IHC-positive area (%) for kidney 4-HNE-Positive (**D**), 8-OHdG-Positive (**E**), RAGE-Positive (**F**), SOD2-Positive (**G**), IHC staining for kidney 4-HNE (**H**), 8-OHdG (**I**), RAGE (**J**), and SOD2 (**K**) in DM rats. NM: normal control; DM: diabetes; DMGL: diabetic plus GLH (low dose: 250 mg); DMGH: diabetic plus GLH (high dose: 500 mg); DMM: diabetic plus metformin. Data are presented as mean ± standard error of the mean (SEM, *n* = 6 per group). * *p* < 0.05 vs. NM, ^†^ *p* < 0.05 vs. DM. Individual data points are represented by circles (NM), squares (DM), upward triangles (DMGL), downward triangles (DMGH), and diamonds (DMM).

To investigate the potential of GLH in regulating renal oxidative stress in DM rats, we assessed oxidative stress-related biomarkers. As depicted in Figure 3, compared to the NM group, the DM group showed significantly elevated levels of renal cortical MDA and positive staining of 4-HNE, 8-OHdG, RAGE, and SOD2 in the kidneys (*p* < 0.05) (Figure 3A,D–K), while PKCα expression remained relatively unchanged (Figure 3C), indicating the presence of renal oxidative damage in DM rats. There was no statistically significant difference in renal AT_1_R expression among the groups (Figure 3B). In comparison to the DM group, there was a notable decrease in renal redox status in the DMGL, DMGH, and DMM groups (*p* < 0.05). All treatments reduced renal 8-OHdG expression compared with DM. The 8-OHdG level in DMGL and DMM did not differ significantly from that in NM, whereas it remained significantly elevated in DMGH compared to the NM group. Renal PKCα expression was significantly reduced in DMGL and DMM, but not in DMGH, compared with DM. These findings indicate marker-specific responses rather than a consistent dose-dependent effect of GLH on renal oxidative injury.

### 2.4. The Effect of GLH on Renal Inflammation and Fibrosis in Rats with Diabetes

**Figure 4:**
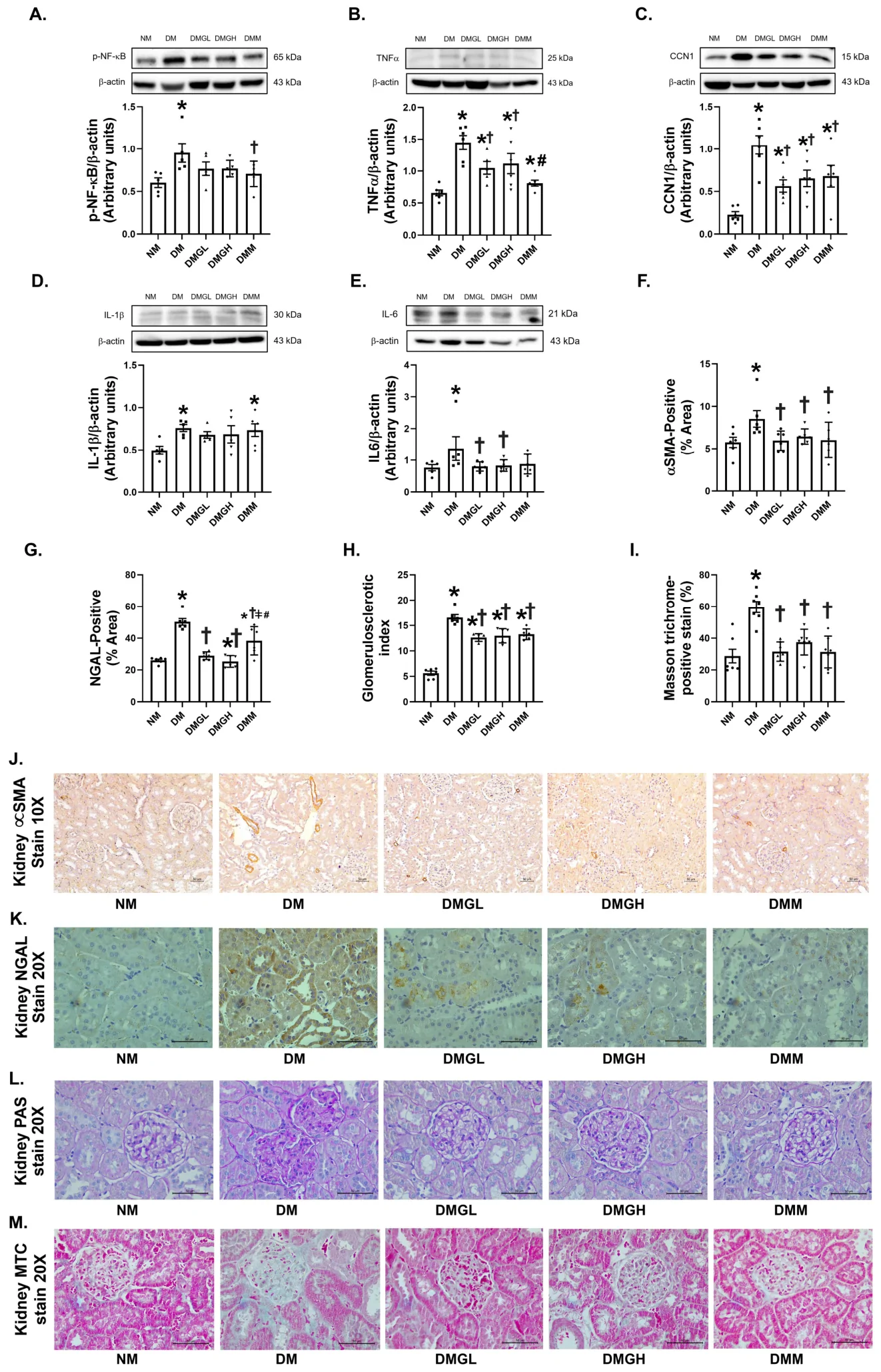
Effects of GLH on renal inflammation and fibrosis. Effects of GLH on the kidney expression levels of p-NF-κB (**A**), TNFα (**B**), CCN1 (**C**), IL-1β (**D**), and IL6 (**E**), IHC-positive area (%) for kidney αSMA-Positive (**F**) and NGAL-Positive (**G**), glomerulosclerotic index (**H**), MTC-positive area (%) (**I**), IHC staining for kidney αSMA (**J**) and NGAL (**K**), PAS stain (**L**), and MTC stain (**M**) in DM rats. The representative gel blots of β-actin in (**D**) and Figure 5G are the same because these bands are the results of the reprobing technique. The representative gel blots in (**D**) (IL-1β, MW 30 kDa) and Figure 5G (Calpain 2, MW 78 kDa) are from the same membrane. Data are presented as mean ± standard error of the mean (SEM, *n* = 5–6 per group). NM: normal control; DM: diabetes; DMGL: diabetic plus GLH (low dose: 250 mg); DMGH: diabetic plus GLH (high dose: 500 mg); DMM: diabetic plus metformin. * *p* < 0.05 vs. NM, ^†^ *p* < 0.05 vs. DM, ^‡^ *p* < 0.05 vs. DMGL, ^#^ *p* < 0.05 vs. DMGH. Individual data points are represented by circles (NM), squares (DM), upward triangles (DMGL), downward triangles (DMGH), and diamonds (DMM).

To investigate the anti-inflammatory and anti-fibrotic effects of GLH on renal tissue in DN, we conducted further analysis of the protein expression of NF-κB and NF-κB-targeted proteins. Western blot results revealed a significant increase in the expression of the proteins p-NF-κB, TNF-α, CCN1, IL-1β, and IL6 in the kidneys of the DM group compared to the NM group (*p* < 0.05) (Figure 4A–E). We also examined the expression of renal fibrosis and injury markers, including α-SMA and NGAL, using immunohistochemistry. The intensity of positive immunostaining was significantly higher in the DM group compared to that in the NM group (*p* < 0.05) (Figure 4F,G,J,K). For histological evaluation of pathological changes, renal sections were stained with PAS and MTC. Compared to the NM group, the DM group exhibited accumulation of glycogen in the glomerular capillary basement membrane and collagen in the interstitial area of the kidneys (*p* < 0.05) (Figure 4H,I,L,M), indicative of severe renal fibrosis. Treatment with metformin significantly decreased the renal protein expression of p-NF-κB (*p* < 0.05), while the DMGL and DMGH groups showed a tendency to reduce this parameter. The expression of the proteins TNF-α, CCN1, and IL6, the positive staining of α-SMA and NGAL, and both PAS and MTC staining were dramatically decreased in the DMGL and DMGH groups compared to the DM group (*p* < 0.05). Interestingly, the DMM group showed a significant decrease in the expression of the renal protein TNF-α compared to that in the DMGH group (*p* < 0.05) and positive staining of NGAL compared to that in the DMGL and DMGH groups (*p* < 0.05). These findings suggest that both low- and high-dose GLH attenuated renal inflammation and fibrosis in DM rats.

### 2.5. The Effect of GLH on Renal ER Stress in Rats with Diabetes

**Figure 5:**
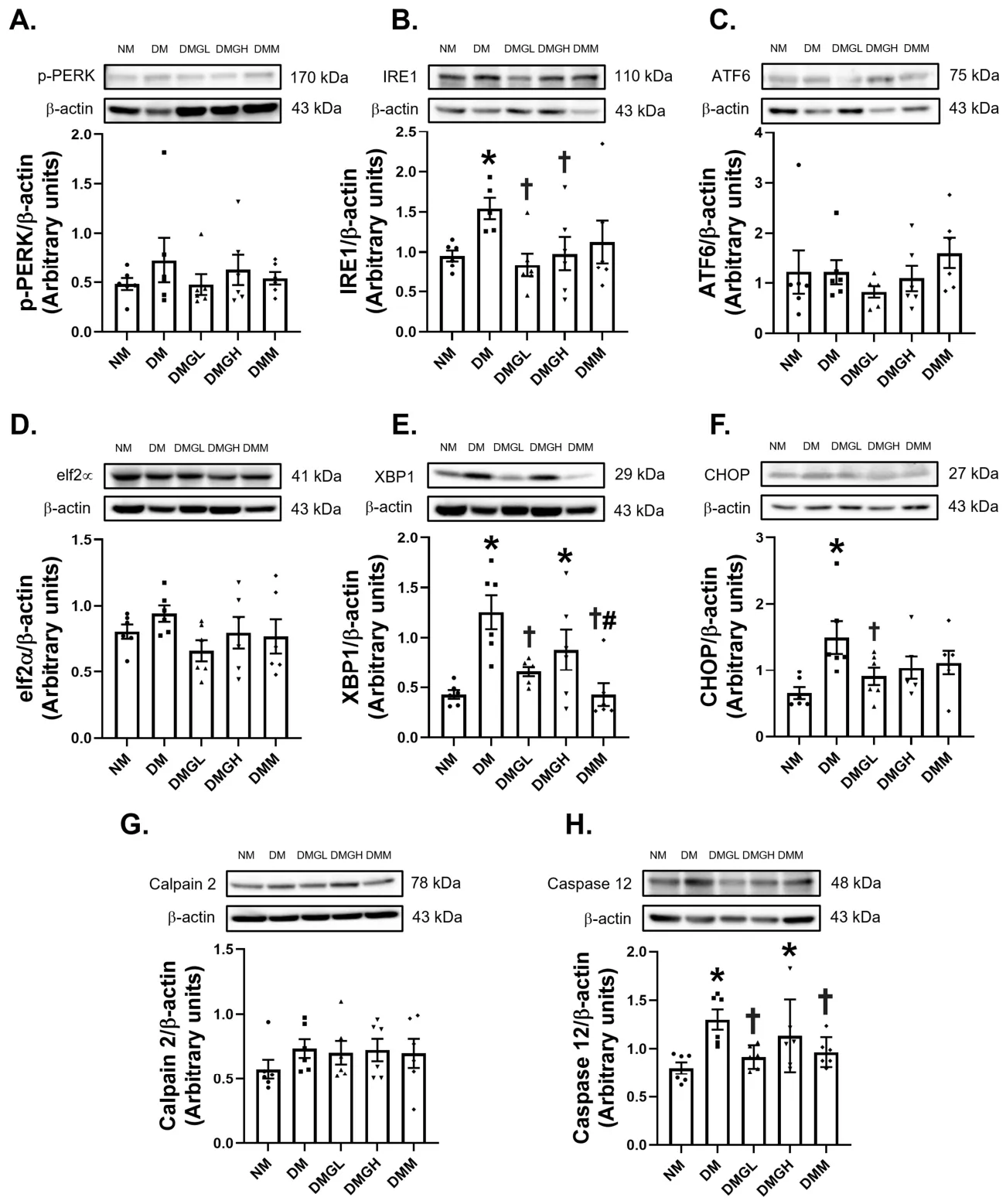
Effects of GLH on renal ER stress. Effects of GLH on the kidney expression levels of p-PERK (**A**), IRE1 (**B**), ATF6 (**C**), elf2α (**D**), XBP1 (**E**), CHOP (**F**), Calpain 2 (**G**), and Caspase 12 (**H**) in DM rats. NM: normal control; DM: diabetes; DMGL: diabetic plus GLH (low dose: 250 mg); DMGH: diabetic plus GLH (high dose: 500 mg); DMM: diabetic plus metformin. The representative gel blots of β-actin in Figure 4D and (**G**) are the same because these bands are the results of the reprobing technique. The representative gel blots in Figure 4D (IL-1β, MW 30 kDa) and (**G**) (Calpain 2, MW 78 kDa) are from the same membrane. The representative gel blots of β-actin in (**D**,**E**) are the same because these bands are the results of the reprobing technique. The representative gel blots in (**D**) (elf2α, MW 41 kDa) and (**E**) (XBP1, MW 29 kDa) were obtained from the same membrane. Data are presented as mean ± standard error of the mean (SEM, *n* = 5–6 per group). * *p* < 0.05 vs. NM, ^†^ *p* < 0.05 vs. DM, ^#^ *p* < 0.05 vs. DMGH. Individual data points are represented by circles (NM), squares (DM), upward triangles (DMGL), downward triangles (DMGH), and diamonds (DMM).

As shown in Figure 5A,C,D,G, there were no significant differences in the expression of renal p-PERK, ATF6, eIF2α, and Calpain 2 among the experimental groups. However, the expression of the renal proteins IRE1, XBP1, CHOP, and caspase 12 was augmented in the DM group compared to the NM group (*p* < 0.05) (Figure 5B,E,F,H). Both the DMGL and DMGH groups showed effective restoration of the renal protein levels of IRE1 (*p* < 0.05), which showed a tendency to decrease in the DMM group compared with the DM group. This further supports the idea that the mitigation of upstream signaling of the ER stress response is involved in the ameliorating effect of GLH in DM rats. Compared with the DM group, renal XBP1 and caspase-12 expression were significantly reduced in the DMGL and DMM groups; however, there was no significant difference in the DMGH group. However, both the DMGL and DMM groups showed a significant reduction in the expression of the renal proteins XBP1 and Caspase 12 compared with the DMM group (*p* < 0.05). These findings indicate that GLH treatment was linked to reduced expression of selected proteins related to IRE1/XBP1 signaling in the kidneys of DM rats.

## 3. Discussion

The major findings of this study are as follows: (1) the administration of GLH is effective in improving metabolic parameters, with the 500 mg dose showing greater effectiveness than the 250 mg dose; (2) parameters pertinent to kidney injury and dysfunction were predominantly improved by the 500 mg dose of GLH; (3) both doses of GLH potentially decreased oxidative injury, inflammation, and fibrosis in the kidney, though not with greater efficacy than metformin therapy; and (4) GLH, particularly at the lower dose, partially attenuated diabetes-associated renal ER stress, as indicated by reduced expression of selected proteins related to the IRE1/XBP1/CHOP/caspase-12 pathway.

The HFD/low-dose STZ model produced hyperglycemia, dyslipidemia, glucosuria, and increased energy and water intake without a significant change in plasma insulin, a metabolic profile consistent with insulin resistance accompanied by partial pancreatic β-cell dysfunction. Impaired insulin-mediated suppression of adipose tissue lipolysis may contribute to elevated circulating lipids, whereas plasma glucose exceeding the renal reabsorptive threshold promotes glucosuria, osmotic diuresis, and compensatory polydipsia [26,27,28]. Both GLH doses improved several metabolic disturbances, although metformin produced a greater reduction in plasma glucose. These improvements may reduce the systemic glucolipotoxic problem that contributes to the progression of DN. Previous studies have suggested that gelatin-derived peptides may improve glycemic control through DPP-4 inhibition and enhanced GLP-1 activity [25,29,30,31]. However, DPP-4 activity and GLP-1 levels were not assessed in the present study; therefore, the involvement of this mechanism remains hypothetical.

GLH treatment improved metabolic parameters, including plasma glucose, food and energy intake, glucosuria, and dyslipidemia. Previous studies have reported that gelatin-derived peptides may improve glycemic control through DPP-4 inhibition and enhanced GLP-1 activity [29,30,31]. However, DPP-4 activity, GLP-1 levels, AMPK activation, and PI3K/Akt signaling were not assessed in the present study. Therefore, the involvement of these pathways remains hypothetical, and further studies directly evaluating these molecular targets are required.

Renal lipid accumulation is an important contributor to DN progression because excessive lipid deposition promotes lipotoxicity, oxidative stress, inflammation, and fibrosis [32,33]. In the present study, increased serum creatinine, BUN, and urinary protein, together with reduced eGFR and increased kidney injury scores, indicated both functional impairment and structural damage in diabetic kidneys. In particular, proteinuria reflects disruption of the glomerular filtration barrier, whereas reduced eGFR indicates impaired renal filtration. GLH ameliorated these abnormalities, with the 500 mg/day dose producing more consistent improvements in renal functional indices than the 250 mg/day dose. The accompanying reductions in renal lipid accumulation and histological injury further support a renoprotective effect of GLH in this DM rat model. Taken together, these findings indicate that GLH supplementation at 500 mg/day improved renal function, as evidenced by increased eGFR and reduced urinary protein excretion. These results support the renoprotective effects of high-dose GLH in DM rat model.

Persistent hyperglycemia and dyslipidemia can induce renal glucotoxicity and lipotoxicity, thereby promoting ROS generation and AGE formation. These processes amplify RAGE-associated oxidative and inflammatory signaling and contribute to the progression of DN [7,9,32,33]. Consistent with this mechanism, the diabetic kidneys exhibited a coordinated pattern of oxidative injury. Increased MDA and 4-HNE indicates enhanced lipid peroxidation, while 4-HNE may additionally act as a reactive aldehyde that modifies cellular proteins and influences redox-sensitive stress and inflammatory signaling. Increased 8-OHdG reflects oxidative DNA damage, whereas elevated RAGE expression may sustain a positive feedback loop between ROS generation and inflammatory signaling. SOD2 is a mitochondrial antioxidant enzyme that converts superoxide into hydrogen peroxide; therefore, its increased expression in the DM group may represent a compensatory response to excessive mitochondrial oxidative stress. AT_1_R and PKCα were measured to assess the possible involvement of angiotensin II–PKC-related redox signaling, which may contribute to ROS production and renal injury. However, their total protein expression did not increase significantly in the DM group, indicating that this pathway was not detectably altered at the protein expression level under the present experimental conditions. Total renal AT_1_R protein expression did not differ significantly among the groups. Because other components of AT_1_R signaling were not assessed, the reason for this unchanged expression cannot be determined from the present data. GLH treatment reduced several oxidative injury markers, suggesting an overall attenuation of the renal oxidative load. The reduction in SOD2 expression after treatment may reflect a decreased requirement for compensatory antioxidant defense. However, the responses were endpoint-specific: the 250 mg/day dose produced greater reductions in certain markers, particularly 8-OHdG and PKCα, whereas the 500 mg/day dose also improved several oxidative injury indices but did not significantly alter PKCα expression. These findings indicate marker-specific responses rather than a consistent dose-dependent effect of GLH on renal oxidative injury. Thus, the findings do not demonstrate a uniform dose-dependent antioxidant effect. Previous studies have reported free radical scavenging and lipid peroxidation-inhibitory activities of gelatin-derived peptides [14,16,17,34,35,36], providing biological context for the observed changes. Nevertheless, the present findings indicate associations with redox-related processes rather than direct modulation of a specific signaling pathway.

Oxidative stress and inflammation form a self-reinforcing association in DN, in which ROS can promote NF-κB activation and cytokine expression, thereby further increasing oxidative injury, renal inflammation, and fibrotic remodeling [37,38,39,40]. Consistent with this interaction, diabetic kidneys exhibited increased p-NF-κB together with elevated TNFα, IL-1β, IL6, and CCN1 expression. The concurrent elevation of NGAL, a marker of tubular injury, and αSMA, an indicator of myofibroblast activation, was accompanied by increased PAS-detected glomerulosclerosis and MTC-detected interstitial collagen deposition. This coordinated pattern indicates that inflammatory activation occurred alongside tubular injury and fibrotic remodeling. Both GLH doses reduced TNFα, CCN1, IL6, NGAL, and αSMA expression and attenuated the pathological changes detected by PAS and MTC staining. Although metformin reduced renal NGAL staining relative to that in the untreated DM group, staining remained higher than in both GLH-treated groups. This may reflect residual tubular injury associated with renal hemodynamic stress or hypoxia [41]. Because these factors were not evaluated, this explanation remains hypothetical. However, neither GLH dose significantly reduced p-NF-κB expression; a significant reduction was observed only in the metformin-treated group. Therefore, the reductions in inflammatory and fibrotic markers following GLH treatment cannot be interpreted as evidence of direct NF-κB inhibition. Moreover, IL-1β expression remained unchanged after treatment, possibly because its maturation requires additional inflammasome-mediated processing that was not evaluated in this study. Unlike TNF-α and IL6, IL-1β requires NF-κB-dependent priming and inflammasome-mediated Caspase-1 processing [42]. Thus, total IL-1β expression may not parallel that of p-NF-κB. However, because inflammasome activation and mature IL-1β were not assessed, this explanation remains hypothetical. Overall, the findings support an association between GLH treatment and reduced renal inflammatory, injury, and fibrotic burden; however, direct modulation of NF-κB or inflammasome signaling was not evaluated in the present study.

Sustained oxidative and inflammatory stress can disrupt ER protein-folding homeostasis and activate the UPR, which comprises three principal signaling branches, initiated by IRE1, PERK, and ATF6. Although the UPR initially attempts to restore ER function, prolonged or unresolved activation may promote inflammation, fibrosis, apoptotic injury, and progressive renal dysfunction in DN [43,44,45,46]. IRE1 is an ER stress sensor whose RNase activity promotes XBP1 mRNA splicing, thereby regulating genes involved in protein folding and ER quality control. In DM kidneys, increased IRE1 and XBP1 protein expression is therefore consistent with an alteration of IRE1/XBP1-related ER stress signaling. However, because IRE1 phosphorylation, RNase activity, and XBP1 mRNA splicing were not evaluated, these changes cannot confirm functional activation of this pathway. Increased CHOP, a stress-associated transcription factor, and Caspase 12, an ER-associated apoptotic protein, may further indicate a shift from an adaptive response toward maladaptive ER stress and cellular injury. In contrast, p-PERK, ATF6, total eIF2α, and Calpain 2 remained unchanged among the groups, indicating that the measured ER stress response was not uniformly altered across all UPR-related proteins. Moreover, because eIF2α phosphorylation and ATF6 cleavage or nuclear translocation were assessed, the functional activity of these branches cannot be determined from the present data. GLH treatment reduced IRE1 and selected downstream ER stress-related proteins, and these changes occurred alongside reductions in oxidative injury, inflammation, renal injury, and fibrosis. Because oxidative and inflammatory stress can interact with ER stress, these coordinated changes are consistent with an overall reduction in renal cellular stress following GLH treatment. Nevertheless, the study assessed only endpoint protein expression and did not include pathway-specific inhibition, knockdown, or functional validation. Therefore, the findings support a relation between GLH treatment and attenuation of selected IRE1/XBP1-related ER stress markers but do not establish direct pathway inhibition or causality. Further functional studies are required to determine whether IRE1/XBP1 signaling is involved in the renoprotective effects of GLH.

**Figure 6:**
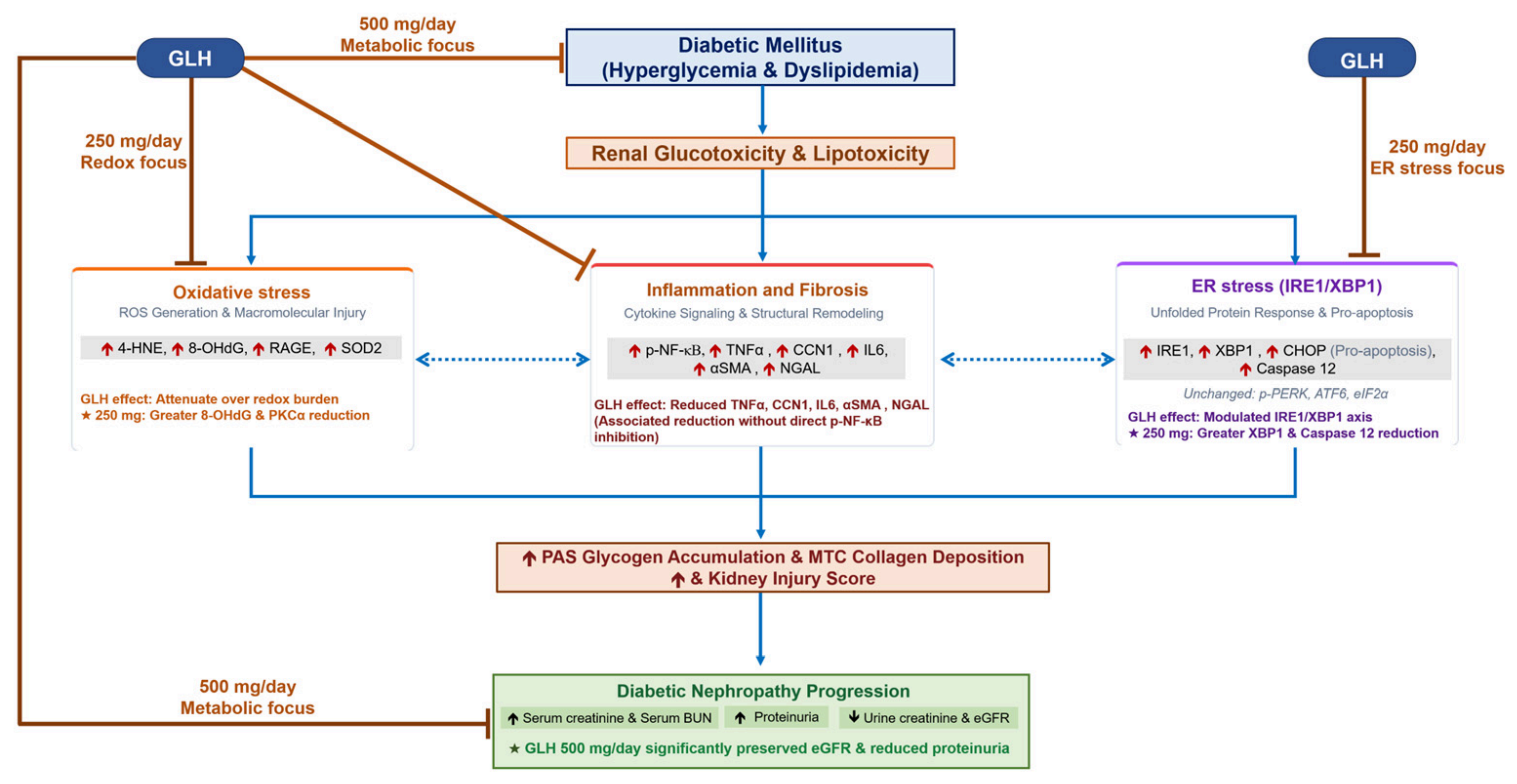
Schematic overview of the possible relationships among renal glucolipotoxicity, oxidative stress, inflammation and fibrosis, ER stress, and DN progression, together with the endpoint-specific effects associated with GLH treatment. The 500 mg/day dose more consistently improved metabolic and renal functional outcomes, whereas the 250 mg/day dose produced greater reductions in selected oxidative and ER stress-related markers. The illustrated relationships represent possible associations rather than confirmed mechanisms. Solid blue arrows indicate the proposed progression of diabetes-related renal injury, dotted bidirectional arrows indicate possible interrelationships among the measured pathways, and brown blunt-ended lines indicate attenuation associated with GLH treatment. Upward and downward arrows denote increases and decreases, respectively. Stars highlight dose-specific findings.

The responses to GLH differed according to the evaluated outcome rather than following a uniform dose-dependent pattern. The 500 mg/day dose produced more consistent improvements in metabolic parameters and renal function, whereas the 250 mg/day dose resulted in greater reductions in certain oxidative stress and ER stress-related markers. These results may reflect the sensitivity of individual biological endpoints to GLH; however, the underlying explanation remains uncertain because only two fixed doses were evaluated and no pharmacokinetic or bioavailability data were obtained. Further studies to identify the optimal effective dose and pharmacokinetics of GLH are required. The measured alterations and their potential relationships are summarized schematically in Figure 6.

## 4. Materials and Methods

### 4.1. Fish Gelatin Hydrolysate Preparation

GLH was prepared using the method described by Wangtueai et al. (2020) [47]. Bigeye snapper (*Priacanthus tayenus*) skins (byproducts obtained from the fish filet and fish mince processing plant in Samut Sakhon Province, Thailand) was used for fish gelatin extraction. In brief, fish skin was washed in tap water, cut into small pieces by hand, and soaked in 5 mL (*v*/*w*) of 0.2 M NaOH solution at 4 °C 3 times, with a new solution each time. The alkali-pretreated skin was washed in running tap water until the pH became neutral, immersed in 5 mL (*v*/*w*) of 0.05 M acetic acid solution for 3 h, and re-washed under running tap water until the pH became neutral. Fish skin gelatin was extracted using distilled water at a 2:1 (*v*/*w*) ratio at 50 °C for 12 h. The extracted solution was passed through two layers of cheesecloth followed by filter paper (No.1, Whatman, Maidstone, UK) and freeze-dried (GFD-3H freeze-dryer, Gririanthong, Ratchaburi, Thailand) to obtain fish skin gelatin. For the preparation of fish skin gelatin hydrolysate, 5% (*w*/*v*) fish skin gelatin was heated at 55 °C, papain (Merck KGaA, Darmstadt, Germany) was added at 3% (*w*/*w* protein), and the mixture was incubated for 3 h to hydrolyze. The mixture was boiled at 95 °C for 10 min to terminate the enzyme reaction, cooled in cold water, and freeze-dried to obtain fish skin gelatin hydrolysate powder.

This study used bigeye snapper skin gelatin hydrolysate, prepared by hydrolyzing bigeye snapper skin gelatin with 3% papain at 55 °C for 3 h, following the method of Wangtueai et al. (2020) [47]. The GLH yield was 69.36 ± 0.49%, with a degree of hydrolysis (DH) of 57.85 ± 0.82%. The crude GLH showed strong DPP-IV-inhibitory activity (IC50: 2.45 ± 0.02 mg/mL) and antioxidant activity, with IC50 values for DPPH, ABTS, OH, and H_2_O_2_ scavenging activities at 2.38 ± 0.01, 1.99 ± 0.03, 0.25 ± 0.01, and 17.64 ± 0.37 mg/mL, respectively. These activities may reflect increased bioactive peptides associated with the higher DH achieved, consistent with Jindapon et al. (2026) [48], who reported that bigeye snapper skin gelatin hydrolyzed with alcalase or papain achieved a higher DH and increased antioxidant and DPP-IV-inhibitory activities. GLH contained high levels of amino acids such as Gly, Ala, Pro, Hyp, Asp, Glu, and Arg. Jindapon et al. (2026) [49] observed that ultrafiltration fractionation increased antioxidant activity, particularly in peptide fractions < 3 kDa. Meanwhile, DPP-IV inhibition appeared to depend more on specific peptide sequences than on molecular weight alone. The peptides identified were primarily composed of hydrophobic and aromatic amino acids, which are often associated with antioxidant and DPP-IV-inhibitory effects.

### 4.2. Experimental Design

Thirty healthy male Wistar rats (6–8 weeks old, 180–200 g) were obtained from Nomura Siam International Corporation in Bangkok, Thailand. They were housed in sanitized cages at the Laboratory Animal Facility, Faculty of Medicine, Chiang Mai University, with a one-week acclimatization period and free access to food and water. All animal experiments were conducted in accordance with the National Research Council’s Guide for the Care and Use of Laboratory Animals, the ARRIVE guidelines, and the U.K. Animals (Scientific Procedures) Act 1986 and associated guidelines. The experimental protocol was reviewed and approved by the Institutional Animal Care and Use Committee of the Faculty of Medicine, Chiang Mai University, Thailand (Permit No. 02/2566). After acclimatization, rats were randomly assigned to two dietary groups: a normal group (NM, *n* = 6) receiving standard chow and a high-fat diet group (HFD, *n* = 24) fed an HFD for four weeks. Type 2 diabetes mellitus (T2DM) was induced in HFD-fed rats by an intraperitoneal injection of 30 mg/kg streptozotocin (STZ) in citrate buffer (400 μL), while NM rats received only the citrate buffer. On the seventh day after STZ administration, fasting blood glucose (FBG) was measured, and rats with FBG > 11.1 mmol/L were confirmed as DM. The DM rats were randomly assigned to five subgroups (*n* = 6 each) using a computer-generated sequence:

Group 1: Normal (NM);
Group 2: Diabetes (DM);
Group 3: Diabetes + GLH (low dose: 250 mg) (DMGL);
Group 4: Diabetes + GLH (high dose: 500 mg) (DMGH);
Group 5: Diabetes + Metformin (100 mg/kg body weight) (DMM).

Due to limited data on the dosage of GLH in DM rat models, the treatment duration and dosage were adjusted based on a previous study that had demonstrated the benefits of Atlantic salmon skin GLH in enhancing insulin secretion and improving glycemic control in DM rats [29]. Atlantic salmon skin GLH at 300 mg/day enhances insulin secretion and glycemic control in DM rats by improving glucose tolerance, inhibiting plasma DPP-4 activity, increasing active glucagon-like peptide-1 (GLP-1) levels, and reducing glucagon levels [29]. Recently, GLH from bigeye snapper (*Priacanthus tayenus*) skin at doses of 250 and 500 mg was shown to exert neuroprotective effects against oxidative stress-related neuronal damage in rats with CCH-induced cognitive impairment [23]. In our study, we selected low (250 mg) and high (500 mg) doses of GLH from bigeye snapper (*Priacanthus tayenus*) skin, focusing not only on metabolic control but also on examining renal alterations and cellular signaling pathways, including redox status, inflammation, fibrosis, and ER stress in DM rats. This fixed-dose regimen (250 and 500 mg/day) was selected based on previous studies in DM rat models demonstrating that comparable daily doses of GLH effectively improve glucose tolerance through the inhibition of plasma DPP-4 activity, which increases active glucagon-like peptide-1 (GLP-1) levels. This results in the enhancement of insulin secretion and a reduction in glucagon levels, ultimately leading to improved glycemic control without adverse metabolic effects, thereby supporting the biological efficacy and safety of a fixed daily dosing approach [29,30]. Metformin, a first-line oral antidiabetic agent, served as a positive control and was obtained from S. Charoen Bhaesaj Trading Company Limited in Bangkok. The selected dose of metformin used in this study was determined based on our previous research [26]. GLH and metformin were freshly dissolved in 0.9% normal saline and given orally via gavage for eight weeks. Weekly recordings of body weight and food and energy intake were made until the end of the experimental period.

After eight weeks of treatment, rat urine volume and water intake were collected using metabolic cages. Rats were then euthanized with isoflurane overdose (>3% *v*/*v*) until reflexes ceased. Blood was drawn from the abdominal aorta, left to clot for 30 min, centrifuged, and stored at −20 °C. Visceral fat and kidneys were weighed and preserved at −80 °C. Kidney histology was assessed by fixing tissue in 10% formalin for 24 h, followed by paraffin embedding. The experimental protocol is illustrated in Figure 1A.

### 4.3. Biochemical Assessments

After euthanasia, whole blood was drawn from the abdominal aorta and placed into tubes containing either sodium fluoride (NaF) or ethylene diamine tetraacetic acid (EDTA). These blood samples were allowed to coagulate and then centrifuged to obtain clear sera for further analysis. Plasma triglycerides, cholesterol, glucose, and urine glucose concentrations were determined using a colorimetric assay kit (Erba Mannheim, Mannheim, Germany). Serum fasting insulin concentration was measured using an Enzyme-Linked Immunosorbent Assay (ELISA kit) from Millipore (Millipore, Burlington, MA, USA), following the manufacturer’s instructions. High-density lipoprotein cholesterol (HDL), low-density lipoprotein cholesterol (LDL), creatinine, and blood urea nitrogen (BUN) in the serum, as well as creatinine in the urine, were analyzed using an automated analyzer at the Associated Medical Sciences Clinical Service Center, Chiang Mai University. The estimated glomerular filtration rate (eGFR) was calculated using the standard equation:

eGFR(ml/min) = (Urine creatinine × Urine flow rate)/Serum creatinine

### 4.4. Assessment of Urinary Protein Levels

Twenty-four-hour urine samples were collected from rats at the end of the experimental period using metabolic cages. The collected samples were clarified by centrifugation at 10,000× *g* for 10 min at 4 °C, and the resulting supernatants were preserved at −20 °C until analysis. Urinary protein concentrations were quantified using a commercial Bradford colorimetric assay kit (Bio-Rad, Hercules, CA, USA), with absorbance measured at 595 nm, and results were expressed as mmol/L.

### 4.5. Assessment of Kidney Lipid Peroxidation

Briefly, a portion of kidney cortex tissues was immersed in a lysis buffer solution containing a protease inhibitor (Roche Applied Science, Indianapolis, IN, USA). After homogenization and centrifugation, the malondialdehyde (MDA) content in the supernatant was assessed using a Thiobarbituric Acid Reactive Substance assay kit (Cayman Chemical Company, Ann Arbor, MI, USA). Total protein content was determined using the Bradford protein assay protocol (Bio-Rad, Hercules, CA, USA). The MDA-to-total-protein ratio was then quantified in nmol/mg protein [39].

### 4.6. Assessment of Kidney Lipid Content

The lipid extraction from kidney tissue followed the protocol outlined by Jaikumkao et al. 2018 [40]. A segment of kidney cortical tissue was homogenized in isopropanol and then centrifuged. The resulting supernatant was used to measure kidney lipid content using a colorimetric assay with a commercial kit (Erba Mannheim, Mannheim, Germany). The lipid content was expressed as mg/g tissue.

### 4.7. Western Blot

Western blot analysis was used to assess proteins associated with diabetes-related redox signaling, inflammation, fibrotic remodeling, and ER stress. Specifically, AT_1_R and PKCα were evaluated as proteins associated with diabetes-related redox signaling; p-NF-κB, TNFα, IL1β, and IL-6 as markers of inflammatory responses; and CCN1 as a marker of fibrotic remodeling. The ER stress response was assessed using p-PERK, IRE1, and ATF6 as UPR sensors; eIF2α and XBP1 as downstream mediators; and CHOP, Calpain-2, and Caspase 12 as proteins associated with ER stress-related apoptosis.

In summary, tissue samples were weighed and homogenized in lysis buffer containing a 1% complete protease inhibitor cocktail (Roche Applied Science, USA). Total protein content was determined using the Bradford protein assay (Bio-Rad, USA). Subsequently, protein samples underwent separation via sodium dodecyl sulfate–polyacrylamide gel electrophoresis (Bio-Rad Laboratories Ltd., Watford, UK), followed by transfer to polyvinylidene fluoride membranes (GE Healthcare, Buckinghamshire, UK). These membranes were then incubated with various primary antibodies, including anti-Angiotensin 2 type 1 receptor (AT_1_R), anti-IRE1, anti-ATF6, anti-X-box binding protein 1 (XBP1), anti-Caspase 12 (Abcam, Waltham, MA, USA), anti-Protein kinase C alpha (PKCα), anti-phosphorylated-Nuclear factor kappa-light-chain-enhancer of activated B cells (p-NF-κB), anti-p-PERK (Cat. #3179, 1:750), anti-Eukaryotic initiation factor 2 alpha (eIf2α), anti-CHOP, anti-Calpain 2, anti-Tumor necrosis factor alpha (TNFα) (Cell Signaling Technology, Danvers, MA, USA), anti-CCN family member 1 (CCN1), anti-Interleukin-1 beta (IL-1β) (Millipore, Burlington, MA, USA), and anti-Interleukin 6 (IL6) (Thermo Fisher Scientific, Waltham, MA, USA). Sodium–Potassium ATPase (Na-K ATPase) (Cell Signaling Technology, Danvers, MA, USA) and β-actin (Millipore, Burlington, MA, USA) served as cellular markers for the membrane fraction and loading control, respectively. After incubation with secondary antibodies, immunoblots were visualized using enhanced chemiluminescence (Bio-Rad, Hercules, CA, USA). Subsequently, protein bands were probed using the iBright™ Imaging System (Thermo Fisher Scientific, Waltham, MA, USA), and protein band densities were quantified using ImageJ 1.50i software from the Research Services Branch (RSB) of the National Institute of Mental Health (NIMH, Bethesda, MD, USA).

### 4.8. Immunohistochemical Staining

Immunohistochemical (IHC) staining was used to assess the tissue distribution of oxidative damage, antioxidant defense, inflammatory signaling, fibrotic remodeling, and tubular injury. Specifically, 4-HNE and 8-OHdG were evaluated as markers of lipid peroxidation and oxidative DNA damage, respectively; SOD2 as an indicator of mitochondrial antioxidant defense; RAGE as a mediator of oxidative and inflammatory signaling; α-SMA as a marker of fibrotic remodeling; and NGAL as a marker of renal tubular injury.

After deparaffinization and rehydration, the sections underwent antigen retrieval, followed by incubation with hydrogen peroxide. Permeabilization with Triton X-100 and blocking using 5% fetal bovine serum were then performed. Next, the sections were incubated overnight at 4 °C with primary antibodies, including anti 4-Hydroxynonenal (4-HNE) (Abcam, Waltham, MA, USA), anti-8-Hydroxydeoxyguanosine (8OHdG) (Bioss, Woburn, MA, USA), anti-Superoxide dismutase 2 (SOD2), anti-Alpha smooth muscle actin (αSMA) (Cell Signaling Technology, Danvers, MA, USA), anti-Receptor for advanced glycation end products (RAGE) (Sigma Aldrich, St. Louis, MO, USA), and anti-Neutrophil gelatinase-associated lipocalin (NGAL) (Millipore, Burlington, MA, USA). Following this, slides were treated with secondary antibodies labeled with horseradish peroxidase for 1 h in a moisture chamber. Corresponding secondary antibodies and 3,3′-Diaminobenzidine (DAB) (HiMedia Laboratories, Thane, India) staining solution were utilized to detect immunoreactivity. The visualization was carried out utilizing a Leica light microscope (Leica Microsystems, Wetzlar, Germany). The quantification of the number of positive cells (% area) was performed using ImageJ 1.50i software from the Research Services Branch (RSB) of the National Institute of Mental Health (NIMH, Bethesda, MD, USA).

### 4.9. Histopathological Study

The kidney was dissected in each case, fixed in 10% formalin, and embedded in paraffin. Sections were then sliced from these paraffin-embedded blocks. For histopathological examination, tissue sections were stained using Hematoxylin and Eosin (H&E) for general tissue structure visualization. Periodic Acid-Schiff (PAS) (Sigma Aldrich, St. Louis, MO, USA) was used to highlight glomerular basement membranes and tubular epithelium, and Masson’s trichrome (MTC) staining (Sigma Aldrich, St. Louis, MO, USA) to visualize collagen deposition in kidneys. After staining, the sections were examined, and images were captured using a light microscope for detailed histological analysis at magnifications ranging from 10× to 40× (Leica Microsystems, Wetzlar, Germany).

H&E-stained kidney sections were examined to semi-quantitatively assess the kidney injury score based on the following lesions: tubular dilatation, interstitial infiltration, tubular vacuolization, interstitial fibrosis, tubular desquamation, and pyknotic nuclei [26]. The glomerulosclerotic index was assessed on PAS-stained sections using a semiquantitative score, as outlined in a previous report [50]. Kidney fibrotic area (% positive area) was calculated and visualized as blue staining in MTC-stained sections [51,52].

### 4.10. Statistical Analysis

All data are expressed as the mean ± standard error of the mean (SEM) from a minimum of five to six independent experiments. Group differences were analyzed using one-way ANOVA followed by the LSD post hoc test for parametric variables. Results were considered statistically significant at *p* < 0.05. Statistical analyses were performed using IBM SPSS Statistics for Windows, Version 32 (IBM Corp, Armonk, NY, USA).

## 5. Conclusions

In conclusion, GLH ameliorated metabolic disturbances and renal dysfunction and reduced renal markers of oxidative injury, inflammation, fibrosis, and ER stress in DM rats. However, the two doses produced distinct, endpoint-specific responses rather than a uniform dose-dependent effect. The 500 mg/day dose more consistently improved metabolic and renal functional outcomes, whereas the 250 mg/day dose produced greater reductions in certain oxidative injury and ER stress-related markers. Therefore, the present findings do not establish either dose as the optimal effective dose. These findings provide proof-of-concept evidence supporting the renoprotective potential of GLH in this DM rat model.

### Limitations of the Study

Because only two fixed doses were evaluated and no pharmacokinetic, bioavailability, or peptide absorption analyses were performed, the present study cannot establish a conventional dose–response relationship or identify an optimal effective dose. In addition, the relatively short treatment duration, absence of formal toxicity assessment, and lack of detailed peptide characterization limit the interpretation and translational relevance of the findings. Further peptide characterization, dose-ranging studies, mechanistic validation, toxicity assessment, pharmacokinetic analysis, and translational studies are required before potential clinical or nutritional applications can be considered. The effective and safe dose of GLH for adults requires further dose-ranging, safety, and clinical studies.

## Data Availability

The original contributions presented in this study are included in the article. Further inquiries can be directed to the corresponding author.
